# Metastatic gastric cancer in fit patients—a practical algorithm of treatment sequencing from the Brazilian Group of Gastrointestinal Tumours (GTG)

**DOI:** 10.3332/ecancer.2025.1848

**Published:** 2025-02-13

**Authors:** Renata D’Alpino Peixoto, Gabriel Prolla, Anelisa Kruschewsky Coutinho, Julia Andrade de Oliveira, Virgilio Souza e Silva, Rachel Riechelmann, Juliana Florinda de Mendonça Rego, Victor Hugo Fonseca de Jesus, Rui Fernando Weschenfelder

**Affiliations:** 1 BC Cancer Agency, Vancouver, BC V5Z 4E6, Canada; 2 Oncoclínicas, Porto Alegre 90570-020, Brazil; 3 Clínica AMO (DASA), Salvador 41950-640, Brazil; 4 AC Camargo Cancer Center, São Paulo 01509-001, Brazil; 5 Oncocentro (DASA), Natal 59020-340, Brazil; 6 Oncoclínicas, Florianopolis 88015-020, Brazil; 7 Hospital Moinhos de Vento, Porto Alegre 90035-000, Brazil

**Keywords:** metastatic gastric cancer, gastroesophageal cancer, algorithm

## Abstract

Recent advancements in biomarker-driven therapies have significantly transformed the treatment paradigm for unresectable metastatic gastric cancer (mGC). These innovations, however, have introduced not only issues related to accessibility but also complexities for treating physicians, particularly general oncologists, in selecting the most appropriate treatment for each patient and deciding on the best sequencing strategy. This manuscript presents an algorithm developed by the Brazilian Group of Gastrointestinal Tumours, designed to provide straightforward guidance in the management of unresectable mGC. This algorithm, grounded in evidence for fit patients, aims to streamline therapeutic decision-making in clinical practice, assuming the absence of access and resource constraints.

## Introduction

According to GLOBOCAN 2020, more than 1,000,000 patients are diagnosed with gastric cancer worldwide, representing 5.6% of all malignancies. Due to the subtle symptoms of early stage disease and the low prevalence of regular screening, most patients are diagnosed at advanced stages, at which point a cure is exceedingly rare. GC accounts for 7.7% of all fatalities, making it the third leading cause of cancer mortality in the world [1]. Consequently, metastatic GC (mGC) represents a substantial public health challenge that warrants further attention.

Recent advancements in biomarker-driven therapies have significantly transformed the treatment landscape for mGC [2]. For instance, since the publication of our group's consensus on GC in 2020, several immune checkpoint inhibitors (ICIs), along with zolbetuximab and trastuzumab-deruxtecan, have gained approval in several countries for use in the metastatic setting [3]. However, these developments have introduced not only issues related to access but also complexities for treating physicians, particularly general oncologists, in selecting the most appropriate treatment for each individual patient.

**Figure 1.**
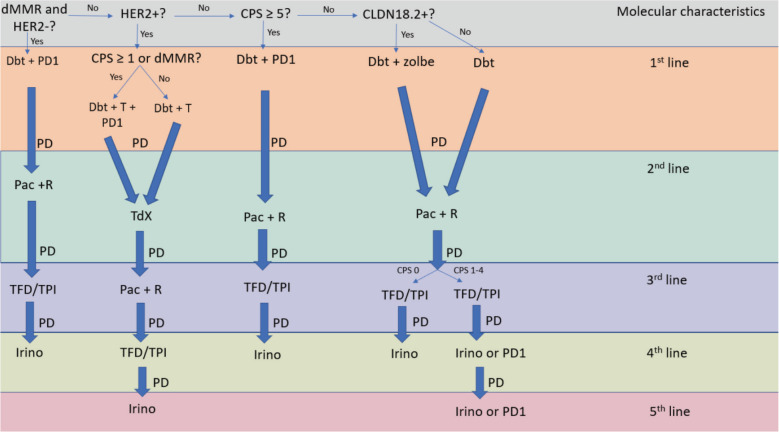
Flowchart describing treatment choices according to molecular features and lines of therapy in mGC.

Unfortunately, most guidelines lack clarity in providing treatment sequencing algorithms for mGC [4–6]. They primarily focus on first-line settings and often fail to incorporate recent advancements. The Brazilian Group of Gastrointestinal Tumours (GTG) acknowledges the challenges in developing straightforward management protocols for unresectable mGC. To address this, GTG proposes an ideal algorithm based on evidence for fit patients to facilitate therapeutic decisions in clinical practice (Figure 1). However, it is important to recognize the limitations of this algorithm in depicting all possible clinical scenarios, including maintenance and locoregional therapies. In addition, we recommend regular assessment and management of symptoms such as pain, nausea and fatigue, as well as nutritional support and psychological counselling for all patients with mGC.

Our algorithm was developed under the assumption that there are no access and resource limitations. Consequently, it should not be regarded as a regulatory guideline. We acknowledge that in many parts of the world, many of the recommended options may not be available. In such cases, one should proceed to the next step in the algorithm. In addition, whenever feasible, patients should be encouraged to participate in clinical trials. For the purpose of this algorithm, gastroesophageal junction adenocarcinomas are considered analogous to GC.

We recommend that all fit patients with mGC be tested for microsatellite instability (MSI)/mismatch repair (MMR), human epidermal growth factor receptor 2 (HER2), programmed cell death ligand 1 (PD-L1) and Claudin 18.2. Stratification based on those current molecular biomarkers offers an opportunity to identify patients who may derive the greatest benefit from the addition of immunotherapy or targeted therapy to a doublet chemotherapy regimen. This regimen typically involves the combination of a fluoropyrimidine (such as 5-fluorouracil or capecitabine) and a platinum agent (such as oxaliplatin or cisplatin). As a group, our preferred chemotherapy doublet regimen is FOLFOX. In many regions globally, S1 is not available and thus will not be considered in our treatment algorithm. The list of relevant molecular features is expected to continue expanding, adding further complexity to the therapeutic strategies for treating mGC.

### Algorithm describing treatment choices according to molecular features and lines of therapy in mGC

The first step in our algorithm involves determining the MSI/MMR status, as patients with MSI-high (MSI-H) or deficient MMR (dMMR) tumours may potentially be cured with ICI. These patients constitute approximately 5% of all mGC cases [7, 8]. Although there is a lack of randomized trials exclusively involving MSI-H/dMMR mGC patients in the first-line setting, data from phase III studies in HER2-negative patients suggest significant benefits from adding anti-programmed cell death 1 (anti-PD1) monoclonal antibodies to chemotherapy. These studies, including CheckMate-649 [9], Keynote-859 [10], Keynote-062 [11], Attraction-4 [12] and ORIENT-16 [13], demonstrate impressive responses and prolonged survival with immunotherapy in the relatively small subgroup of MSI-H/dMMR patients. For instance, the unstratified hazard ratios (HRs) for overall survival (OS) with nivolumab plus chemotherapy versus chemotherapy alone among patients with MSI-H and microsatellite stable (MSS) mGC were 0.37 (0.16–0.87) and 0.80 (0.71–0.91), respectively, across all randomized patients. However, only 3% of these patients had MSI-H tumours [9].

For patients with MSI-H and HER2-negative mGC who progress on chemotherapy combined with an anti-PD1 agent and remain fit for further treatment, we typically offer a combination of paclitaxel and ramucirumab, as supported by the Rainbow phase III trial [14]. Although this trial did not stratify patients by molecular features, those who received paclitaxel and ramucirumab demonstrated a longer median OS compared to those receiving only paclitaxel (9.6 versus 7.4 months, respectively – HR 0.8, *p* = 0.017). Upon progression following the second-line treatment with paclitaxel and ramucirumab, the best evidence and our recommendation is to offer trifluridine-tipiracil (TFD/TPI), based on the findings of the TAGS phase III trial [15]. Median OS was 5.7 months in the TFD/TPI group and 3.6 months in the placebo group (HR 0·69, *p* = 0·00058). In the rare circumstances where a patient progresses on third-line chemotherapy and remains well enough to continue systemic treatment, we would offer irinotecan, despite the low level of evidence supporting its efficacy [16, 17].

Approximately 7%–20% of mGC patients harbour HER2-positive tumours. The epidemiologic LEGACY study, which analysed data from 689 patients with GC across Europe and Latin America (LATAM), reported an HER2 positivity rate of 13.8% among patients from LATAM [18]. Since the publication of the phase III Toga trial, the addition of trastuzumab to a doublet chemotherapy became a standard of care in the first-line setting by increasing median OS from 11.1 to 13.8 months (HR 0.74, *p* = 0.0046) [19]. In our opinion, a chemotherapy doublet with trastuzumab remains the gold standard treatment for those HER2-positive, MSS and PD-L1 negative tumours. For those HER2-positive tumours that are also PD-L1 positive by combined positive score (CPS) ≥1 and/or MSI-H/dMMR, we recommend the addition of an anti-PD1 agent based on the Keynote-811 trial [20]. As presented at the European Society for Medical Oncology 2023 Meeting, the third interim analysis revealed that the addition of pembrolizumab to chemotherapy and trastuzumab increased progression-free survival (PFS) from 8.1 to 10 months (HR 0.72, *p* = 0.0002) [20]. This improvement was even more pronounced among patients with a CPS of 1 or greater, with PFS increasing from 7.2 to 10.8 months (HR 0.70). Furthermore, on 01 May 2024, Merck announced that OS was also significantly increased by the addition of pembrolizumab [21].

When HER2-positive patients progress on a first-line trastuzumab-containing regimen, regardless of their CPS or MSI status, we recommend second-line treatment with trastuzumab deruxtecan based on the results of the phase II DESTINY-Gastric01 and DESTINY-Gastric02 trials [22, 23]. In these studies, overall response rates (RRs) ranged from 42% to 51%, which is superior to the RRs of less than 30% typically achieved with traditional second-line treatment with paclitaxel and ramucirumab. In addition, the median OS was 12.5 months for the group that received trastuzumab deruxtecan, compared to 8.4 months for those who received the investigator’s choice of chemotherapy in the DESTINY-Gastric01 trial [22]. However, it is important to acknowledge that the optimal second-line treatment for patients with HER2-positive tumours who progress following first-line therapy with a chemotherapy doublet plus trastuzumab, with or without pembrolizumab, remains uncertain. Based on current evidence, trastuzumab deruxtecan appears to provide superior RRs and OS benefits when compared indirectly to paclitaxel and ramucirumab in the second-line setting. Furthermore, it is crucial to confirm HER2 positivity at the time of progression on first-line therapy through a biopsy, particularly if trastuzumab deruxtecan is being considered as a therapeutic option. Following failure on trastuzumab deruxtecan, we recommend subsequent treatment with paclitaxel and ramucirumab. If progression continues, the next line of therapy would be TFD/TPI, followed by irinotecan, as previously mentioned.

For patients with MSS and HER2-negative tumours, the next step is to determine their PD-L1 status, despite it being a complex and heterogeneous biomarker [24]. Numerous recent clinical trials of ICIs for the treatment of gastric cancer have reported that the CPS is a valuable predictor of treatment response to ICIs [9–13]. Furthermore, it is estimated that approximately 55%–66% of patients with advanced GC express PD-L1 [9–13]. CPS is defined as the number of cells stained positive for PD-L1 (including tumour cells, lymphocytes and macrophages) as a proportion of the total number of tumour cells, multiplied by 100.

Although various PD-L1 IHC assays have been validated across different solid tumours to guide patient selection for immunotherapy, in GC, the antibodies 22C3 and 28-8 are more commonly utilized in clinical practice. This preference arises from their extensive use in clinical trials assessing pembrolizumab and nivolumab. When CPS is applied, analytical concordance in PD-L1 testing between different PD-L1 assays has been suggested [25].

Many regulatory agencies worldwide have initially approved the use of anti-PD1 agents in combination with chemotherapy regardless of PD-L1 status. However, we believe that patients with a CPS <5 do not derive significant benefit from ICIs. Based on the available evidence, we recommend the use of chemotherapy in conjunction with an anti-PD-1 agent when the CPS is 5 or higher. For CPS levels between 1 and 4, the incorporation of immunotherapy may be considered; however, this should be weighed against the increased costs and potential adverse events, given its relatively modest added benefit. Furthermore, no randomized clinical trials have demonstrated a statistically significant benefit of adding an anti-PD-1 antibody to chemotherapy in patients with CPS scores ranging from 1 to 4 or even from 1 to 10 [9–13].

Following progression or intolerance to chemotherapy and an anti-PD1 agent, we recommend second-line treatment with paclitaxel and ramucirumab. Subsequent lines of therapy would be TFD/TPI followed by either irinotecan or an anti-PD1 agent in monotherapy. The ATTRACTION-2 phase III trial demonstrated that nivolumab modestly improved OS versus placebo in third or later lines of therapy (5.3 versus 4.1 months; HR 0.62, *p* < 0.0001) [26], while the Keynote-059, a single arm phase II trial, demonstrated improved RR with pembrolizumab among those patients with CPS ≥1 [27]. Consequently, an anti-PD-1 agent represents a rational option for third-line or subsequent therapy in cases where the CPS is ≥1, provided that it has not been utilized in prior treatment.

And that brings us to the final part of our algorithm: the status of Claudin 18.2 (CLDN18.2), an emerging and promising target in mGC. Recent data from both the phase III Spotlight and GLOW trials have unveiled the efficacy of zolbetuximab, a CLDN18.2-targeting antibody, in combination with oxaliplatin-based chemotherapy for CLDN18.2-positive metastatic GC. Those studies have demonstrated an OS gain with the addition of zolbetuximab, despite no significant increases in RRs [28, 29]. The median OS reached 18.2 months for patients who received FOLFOX and zolbetuximab, compared to 15.5 months for those who received only chemotherapy, according to the Spotlight trial [28].

For patients who are MSS, HER2-negative, CPS-negative and CLD18.2-negative, we would recommend chemotherapy as the primary treatment option. In the case of a fit patient who requires a strong response, it is worth considering whether offering a triplet regimen (a combination of a fluoropyrimidine, a platinum agent and a taxane) could potentially improve outcomes, although this approach remains debatable since it significantly increases toxicity [30, 31]. Later lines of treatment would follow the previous discussion unless the patient has received a triplet in the first line. For those cases, we would only ramucirumab as a single agent in the second line [32]. Patients who have either received first-line FLOT or experienced relapse within 12 months after perioperative FLOT may consider FOLFIRI in combination with ramucirumab as a viable option to address prior taxane exposure. Although the phase II RAMIRIS trial did not achieve its primary endpoint of OS compared to historical controls, the 25% RR observed in patients previously treated with docetaxel represents a clinically meaningful outcome in this context [33]. Nonetheless, access to the combination of FOLFIRI and ramucirumab remains challenging.

The incorporation of novel biomarkers is anticipated to advance the therapeutic landscape for GC in the near future. Among these, Fibroblast Growth Factor Receptor 2 (FGFR2) has emerged as one of the most promising actionable targets. FGFR2 amplification is observed in approximately 3%–10% of advanced GC cases, with a higher prevalence in diffuse or poorly differentiated subtypes [34, 35]. In the phase II **FIGHT trial**, the addition of **bemarituzumab**, a monoclonal antibody targeting FGFR2b, to FOLFOX chemotherapy demonstrated significant activity in the first-line treatment of patients with FGFR2b overexpression, as identified by IHC, and/or FGFR2 gene amplification detected through circulating tumour DNA analysis [36].

Building on these findings, phase III trials are currently underway to further evaluate bemarituzumab in FGFR2-positive patients. These include the **FORTITUDE-101 trial (NCT05052801)**, assessing its combination with FOLFOX alone, and the **FORTITUDE-102 trial (NCT05111626)**, investigating its addition to a regimen of FOLFOX plus nivolumab. These studies aim to establish the efficacy of FGFR2-targeted therapy in this molecularly defined subset of patients with advanced GC.

Although rare, genetic alterations, such as BRAF mutations, high tumour mutational burden outside of MSI-H status and gene fusions, can occasionally be identified in mGC [32]. These alterations are not currently incorporated into standard treatment algorithms. However, their potential therapeutic relevance is acknowledged, and targeted therapies may offer benefits for this subset of patients. When accessible, comprehensive genomic sequencing could provide valuable insights to guide treatment decisions in these cases.

## Conclusion

With this algorithm, the Brazilian Group of GTG posits that most scenarios involving unresectable mGC in fit patients are addressed, thereby offering substantial assistance to clinicians in making therapeutic decisions. Nonetheless, it is crucial to consider access and resource limitations in actual clinical practice. Clinical trial enrolment must remain a priority in this setting, given the persistently poor outcomes. In addition, it is imperative to continue gaining molecular insights to better characterize the heterogeneous nature of GC.

## List of abbreviations

CLDN18.2, Claudin 18.2; CPS, Combined positive score; Dbt, Doublet; dMMR, Deficient mismatch repair; HER2, Human epidermal growth factor receptor 2; Irino, Irinotecan; Pac, Paclitaxel; PD, Progression of disease; PD1, Anti-programmed cell death protein 1 monoclonal antibody; R, Ramucirumab; T, Trastuzumab; TdX, Trastuzumab deruxtecan; TFD/TPI, Trifluridine/tipiracil; Zolbe, Zolbetuximab.

## Conflicts of interest

The authors declare no conflict of interest for this manuscript.

## Funding

None.
